# RETRACTION: Optimization and Modeling of Cr (VI) Removal from Tannery Wastewater onto Activated Carbon Prepared from Coffee Husk and Sulfuric Acid (H_2_SO_4_) as Activating Agent by Using Central Composite Design (CCD)

**DOI:** 10.1155/jeph/9804619

**Published:** 2026-05-20

**Authors:** Journal of Environmental and Public Health

RETRACTION: T. A. Amibo, B. S. Mustefa, A. B. Bayu, and W. F. Kabeta, “Optimization and Modeling of Cr (VI) Removal from Tannery Wastewater onto Activated Carbon Prepared from Coffee Husk and Sulfuric Acid (H_2_SO_4_) as Activating Agent by Using Central Composite Design (CCD),” *Journal of Environmental and Public Health* 2023, no. 1 (2023): 1–14, https://doi.org/10.1155/2023/5663261.

The above article, published online on 30 January 2023 in Wiley Online Library (https://wileyonlinelibrary.com), has been retracted by agreement between the journal Editor, Rahil Changotra and John Wiley & Sons Ltd.

The retraction has been agreed upon following an investigation into concerns raised by a third party and Pubpeer comments [1] regarding image inconsistencies.

The XRD image presented in Figure 3 shows areas of repeating signal patterns (e.g., RCH 35–40° and 60–65°). During the investigation, the raw data were requested; however, the authors were unable to provide any.

The visual similarity of the repeating signal along with the absence of raw data undermines our confidence in the integrity of the article’s content, and it is, therefore, retracted.

The authors were informed and acknowledged the article’s retraction. However, they did not choose to provide their formal agreement or disagreement.
